# Genome-Wide Transcription Analysis of Clinal Genetic Variation in *Drosophila*

**DOI:** 10.1371/journal.pone.0034620

**Published:** 2012-04-13

**Authors:** Ying Chen, Siu F. Lee, Eric Blanc, Caroline Reuter, Bregje Wertheim, Pedro Martinez-Diaz, Ary A. Hoffmann, Linda Partridge

**Affiliations:** 1 Department of Genetics, Evolution and Environment, University College London, London, United Kingdom; 2 Bio21 Institute, Department of Genetics, The University of Melbourne, Parkville, Victoria, Australia; 3 MRC Centre for Developmental Neurobiology, King's College, London, United Kingdom; University of Umeå, Sweden

## Abstract

Clinal variation in quantitative traits is widespread, but its genetic basis awaits identification. *Drosophila melanogaster* shows adaptive, clinal variation in traits such as body size along latitudinal gradients on multiple continents. To investigate genome wide transcription differentiation between North and South that might contribute to the clinal phenotypic variation, we compared RNA expression patterns during development of *D. melanogaster* from tropical northern and temperate southern populations using whole genome tiling arrays. We found that genes that were differentially expressed between the cline ends were generally associated with metabolism and growth, and experimental alteration of expression of a sample of them generally resulted in altered body size in the predicted direction, sometimes significantly so. We further identified the *serpent (srp)* transcription factor binding sites to be enriched near genes up-regulated in expression in the south. Analysis of clinal populations revealed a significant cline in the expression level of *srp*. Experimental over-expression of *srp* increased body size, as predicted from its clinal expression pattern, suggesting that it may be involved in regulating adaptive clinal variation in *Drosophila*. This study identified a handful of genes that contributed to clinal phenotypic variation through altered gene expression level, yet misexpression of individual gene led to modest body size change.

## Introduction

Clinal phenotypes and genotypes vary gradually over geographical space, often as a result of environmental heterogeneity. Many species, including our own, show clinal variation in traits that appear to be of adaptive significance. A particularly striking example is body size variation in *Drosophila melanogaster*. On several continents there is a clinal increase of body size toward higher latitudes [1], [2], [3]. The body size cline on the eastern side of Australia has been particularly well studied. Chromosome substitution and quantitative trait locus (QTL) mapping have shown that most variation in size between cline end populations is controlled by genes located on the right arm of the third chromosome [4], [5]. Association analysis of a population from the center of the cline indicated that body size is associated with *In(3R)Payne*, a chromosomal inversion located on the right arm of chromosome 3 that also varies clinally in frequency [6]. A recent QTL mapping study between cline end populations from the eastern Australian cline has identified two distinct regions within *In(3R)Payne* controlling latitudinal variation in body size [7]. Clinal variation of wing size is also partly associated with variation around the *Dca* gene, located just outside the *In(3R)Payne* inversion [8].

While QTL analysis is useful to identify broad scale chromosomal regions that affect a phenotypic trait, comparative study of transcription variation is potentially a powerful approach to identify individual genes that contribute to complex trait [9]. Changes in the patterns of gene expression are of interest because they are believed to underlie many of the phenotypic differences within and between species [10], [11]. Indeed, an increasing number of empirical studies highlights the pervasive phenotypic effects of regulatory variation both in human and model organisms, for traits such as skeletal morphology in stickleback fish [12], beak morphology in Darwin finches [13], cuticular pigmentation in *Drosophila* [14], and disease susceptibility in humans [15], [16], [17]. Furthermore, *Drosophila melanogaster* provide a tractable system for studying variation in both gene expression and phenotypic traits along a cline, because of the powerful genetic tools available and an extensive history of analysis of spatially varying selection in this species.

Here we report a systematic effort to identify differences in gene expression during development between northern and southern Australian *Drosophila melanogaster* populations, using whole genome expression profiling with the *Drosophila* tiling array. We identified candidate genes that are significantly differentially expressed between the two ends of the cline and carried out detailed molecular and population genetic analysis to evaluate their role in shaping clinal population differentiation in body size. Statistical analysis of enrichment of functional categories among differentially expressed genes revealed that the binding sites of the *srp* transcription factor (TF) were significantly enriched near genes up-regulated in South 2^nd^ instar larvae. Further population survey identified significant clinal variation in expression of the *srp* gene along the latitudinal gradient. Over-expression of *srp* significantly increased wing size, as predicted from its expression cline, suggesting that *srp* may be involved in regulating the clinal size variation in *Drosophila*.

## Results and Discussion

### 1. Differential gene expression between northern and southern populations

To identify genes that are differentially expressed along the body size cline in eastern Australia, we sampled five northern and five southern populations from the extreme ends of the cline (Table S2) and tested for differences in gene expression at two developmental time points, in the second and third instar larval stages, using the *Drosophila* tiling array. Data analysis was carried out using the hierarchical Bayesian model implemented in Limma [18] to infer differential expression between North and South at each developmental time point separately. The procedure identified 826 probes covering 67 genes and 5 intergenic regions in the second instar larval stage, and 777 probes covering 70 genes and 6 intergenic regions in the third instar larval stage. Fourteen genes were differentially expressed in both stages, a much greater degree of overlap than would be expected by chance (P<<0.001), implying both a genuine signal in the data and that some genes are indeed differentially expressed between northern and southern populations during a large part of larval development. There was, however, very little overlap among expression differences for intergenic regions, suggesting that these differences might be largely due to expression noise. DNA sequence variation between North and South might affect transcription analysis (see Material and Methods), which might lessen power to detect differentially expressed genes, and lead to a substantial false discovery rate [19]. This may partially explain why there were relative few differentially expressed genes between North and South.

### 2. Statistical analysis of functional categories

To further identify potentially important biochemical processes in a statistically rigorous way, we made use of the freely available software package Catmap (http://bioinfo.thep.lu.se/Catmap) [20]. This program assigns significance to gene categories based on their relative statistical ranking or representation within the differentially expressed genes, in order to determine if any gene annotation is represented more often than expected by chance. We ran Catmap analysis on the ranked gene list, based on the Bayesian *t* statistics of differentially expressed genes for over-representation of functional categories from a number of biological databases, including Gene Ontology (GO) and InterPro, and several customized databases that contain microarray data and functional classifications from previously published studies. This generated a list of gene categories showing significant over-representation among the genes with altered expression between the cline ends (Table 1). Metabolic and oxidation related processes were significantly up-regulated in the South (Table 1), possibly due to higher metabolic rates at higher latitudes [21]. The gene categories that were over-represented among up-regulated genes in populations from the North, on the other hand, were mostly related to cellular component organization and biogenesis. Chromosome 3R was over-represented in up-regulated genes in the South at both developmental stages, consistent with the mapping of the body size difference between cline ends to this genomic region.

**Table 1:** Process level comparison between North and South.

Functional categories	p value
**Up-regulated in South 2^nd^ instar**	
Chr3R: 10000001–20000000	1.59E-06
*srp* TF binding genes	1.16E-05
BP_GO:0044262 cellular carbohydrate metabolic process	1.52E-05
MF_GO:0016645 oxidoreductase activity, acting on the CH-NH group of donors	5.42E-05
**Up-regulated in North 2^nd^ instar**	
CC_GO:0000119 mediator complex	2.06E-05
BP_GO:0016043 cellular component organization and biogenesis	4.28E-05
BP_GO:0022607 cellular component assembly	9.10E-05
**Up-regulated in South 3^rd^ instar**	
BP_GO:0017038 protein import	1.26E-06
chr3R:10000001–20000000	3.10E-05
MF_GO:0016645 oxidoreductase activity, acting on the CH-NH group of donors	7.97E-05
BP_GO:0008152 metabolic process	1.78E-04
**Up-regulated in North 3^rd^ instar**	
miR-315 (AATCAAA)	1.10E-06
BP_GO:0050877 neurological system process	4.24E-05
BP_GO:0007600 sensory perception	1.01E-04
BP_GO:0016043 cellular component organization and biogenesis	1.11E-04

Significantly up- or down-regulated functional categories at false discovery rate estimated by gene category permutation <0.1 are indicated, with significance determined using Catmap. BP_GO: biological process gene ontology; MF: molecular function; CC: cellular component.doi:10.1371/journal.pone.0034620.t001

The observed gene expression differences between cline ends might be due to the effect of differential activity of transcription factors. The result of Catmap analysis suggested that *srp* TF binding site was significantly over-represented near genes that were up-regulated in the South 2^nd^ instar larvae. The *srp* gene is located at 89A, near the proximal breakpoint of *In(3R)Payne*. It is an endoderm-specific GATA transcription factor essential for fat body and blood cell development, and positively regulates cellular biosynthetic processes and the humoral immune response [22], [23], [24], [25], [26], [27], [28], [29].

### 3. Size effect of the candidate genes by mis-expression

**Figure 1:**
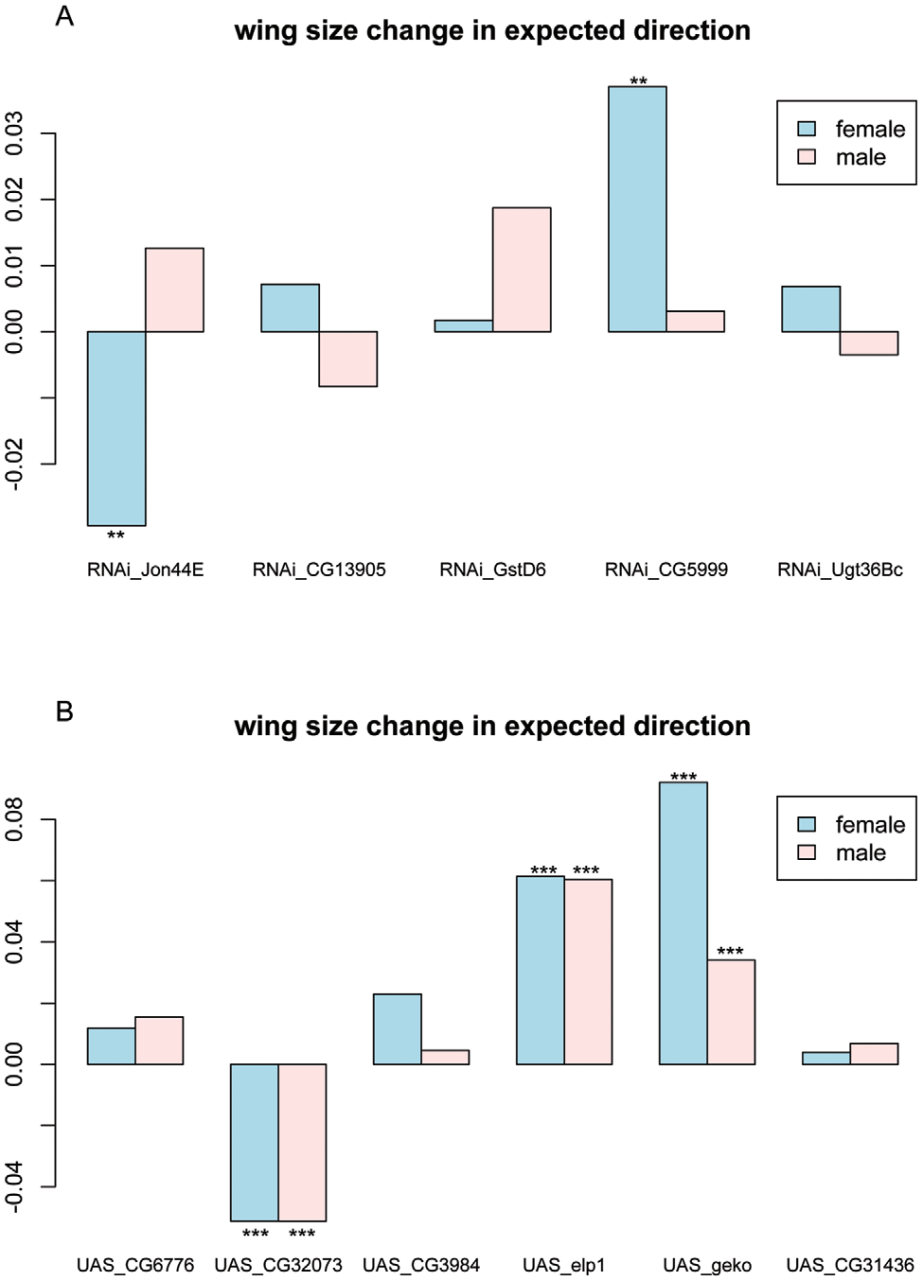
Change in wing size where candidate genes were subjected to (A) RNAi knock down of their expression level; (B) Overexpression. The y-axis indicates the effect of RNAi or overexpression on wing size (mm^2^). Positive measure indicates concordance with the prediction based on their expression profiles. ***P<<0.001 **P<0.01 after Bonferroni correction for multiple comparisons.

We verified the expression differences of the 29 genes with the most significant P values (false discovery rate FDR <0.01) (Table S1) by qRT-PCR with cDNA samples from the population samples used in the tiling array analysis plus an additional 5 northern and 5 southern cline end populations (Table S2 and S3). We confirmed that all of the 29 genes were indeed differentially expressed between northern and southern populations (Table S1). We selected 11 of these candidate genes to experimentally analyse their effect on body size using the GAL4/UAS system. Some of the selected genes (*CG11034, Jon44E, CG6776, elp1, GstD6, CG5999 and Ugt36Bc*) are clearly involved in metabolism, while others (*CG32073, CG3984, CG13905* and *CG31436*) have no known function. For the latter group, this study may help annotate gene function. UAS-RNAi or p{UAS} lines of the 11 candidate genes were individually crossed to a constitutively and ubiquitously expressed driver *daughterless-GAL4* (*da-GAL4*) line. The average wing size (a reliable indicator of body size) of the progeny was compared to that of the control cross offspring (*da-Gal4/+; wDah*). All lines were made co-isogenic by multiple generations of backcrossing and standard density rearing was used to generate flies for wing size measurement. The change in wing size between mis-expression and control crosses are shown in Figure 1. If directions of wing size change were consistent with their expression profile between North and South, they were given a positive value and if contrary a negative value. Mis-expression of most of the candidate genes produced the expected direction of change in wing size, with the exception of *CG32073*, which was up-regulated in the South yet produced smaller wing size in both sexes when over-expressed. However, the effects on wing size were generally small and sometimes inconsistent between females and males, apart from over-expression of *elp1* and *geko* (Figure 1). Over-expression of these two genes led to a striking 5% and 8% decrease in female wing size, suggesting *elp1* and *geko* might indeed be involved in regulating body size, because both are up-regulated in the North where flies are smaller. The *elp1* gene codes for an RNA-dependent RNA polymerase involved in RNAi and transposon suppression [30]. *Geko* is involved in sensory perception of smell and behavioral response to ethanol [31], [32]. There was no obvious link between the function of the two genes and body size. Overall the effect of each individual gene was small, suggesting that the body size cline is controlled by multiple loci, many of them contributing with minor effect.

### 4. Clinal variation of srp expression level and the size effect of srp over-expression

**Figure 2:**
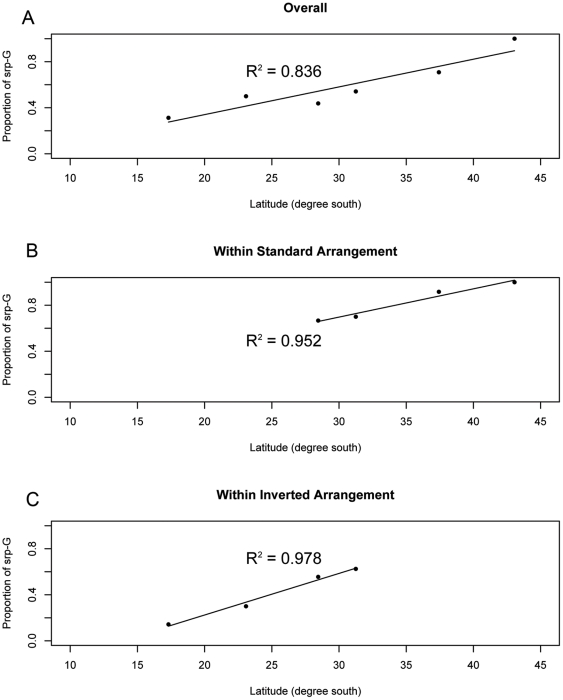
*srp* expression level affect body size. A. Clinal variation of *srp* mRNA level. Open circle indicates outlier Coffs Harbour population. B *srp* over-expression increases wing size: comparison to *da-GAL4/+; wDah* ***P<0.001 after Bonferroni correction for multiple comparisons. *UAS-srp/da-Gal4* showed 10 fold increase, while *UAS-srp/+* showed 5 fold increase of *srp* mRNA level compared to *da-Gal4/+*.

To uncover any clinal variation associated with *srp* that might be important in clinal adaptation of traits, we made an extensive survey of *srp* gene expression level in 15 populations along the east coast of Australia. We detected a positive linear cline of *srp* expression level along this latitudinal gradient (R^2^ = 0.36; P<0.05). The Coffs Harbour population was an outlier (Bonferroni p-values for Studentized residuals P = 0.03) and the latitudinal association was much stronger when this population was excluded (R^2^ = 0.60; P<0.01) (Figure 2A).

Higher expression levels of *srp* at higher latitudes suggests that *srp* might be a positive regulator of body size. This was confirmed by *srp* over-expression, which significantly increased wing size in both sexes (P<0.001) (Figure 2B). There was leaky over-expression of *srp* in the *UAS-srp* line itself as revealed by real time PCR (data not shown), and both the experimental and UAS line control (*UAS-srp/+; wDah*) crosses produced significantly larger wing area than in the Dahomey control (Figure 2B).

### 5. Clinal sequence analysis of the srp gene

**Figure 3:**
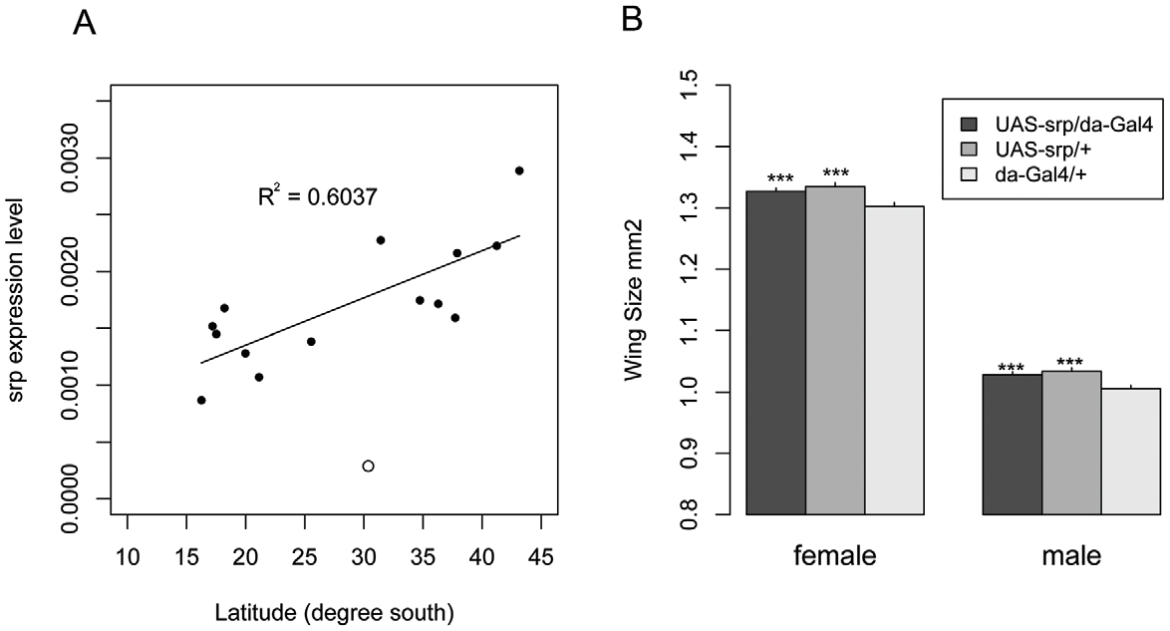
Associations between latitude and frequency of *srp-G* allele in the 2008 field collection from eastern Australia. Linear regressions were significant overall (R^2^ = 0.836, P = 0.00676) and for individuals with the Standard *In(3R)P* chromosome arrangement (R^2^ = 0.952, P = 0.016), and with the Inverted arrangement (R^2^ = 0.978, P = 0.00739).

The clinal expression variation of *srp* might be caused by nearby DNA sequence variation. To investigate this, we sequenced 5′ flanking and N terminal regions of the *srp* gene in 96 isofemale lines originating from six geographical locations along the cline. We did not find any significant clinal sequence variation within the 2kb 5′ flanking region, suggesting *srp* might be under the control of elements that reside elsewhere in the genome be they *cis*-acting or *trans*-acting sequences. We detected a steep cline of a non-synonymous polymorphism (site 8330) at the second exon of the *srp* gene (P = 0.0475, Figure 3A), which seemed to persist within both the standard and inverted chromosomal arrangements (Figure 3B & 3C). However, an association study in a mid-latitude population failed to uncover any correlation between the non-synonymous change and body size (data not shown). It is thus possible that the non-synonymous polymorphism is neutral and hitch-hiking along with another mutation which causes the cline, or that it controls some other clinally varying trait, such as heat resistance, time to recover from a chill-induced coma, or starvation resistance [3], [33], [34].

*Srp* was first identified as a transcriptional activator that binds to the regulatory sequences of the *alcohol dehydrogenase* (*Adh*) gene [22]. There are two common alleles at the *Adh* locus, *Adh^F^* and *Adh^S^*. These present a classic example of clinal variation in gene frequency: the frequency of the fast allele is low in the tropical regions and increases with latitude. It appears, from active-site titration experiments [35], that not only does *Adh^F^* have a higher catalytic efficiency, but that it is also present at higher concentrations. This could potentially be caused by the gradient in *srp* expression that we have described, although it has not been confirmed that the difference in Adh enzyme concentration is due to differences in expression [36], [37].

## Materials and Methods

### Fly strains

Adult flies were collected in 2005 and 2008 from various latitudes along the east coast of Australia (Table S2 and S3). No specific permits were required for the described field studies. Ten isofemale lines from each location were initiated with the progeny of single field-collected females. Two to three generations after collection, a mass-bred (MB) population was founded with 20 males and 20 females from each of the 10 isofemale lines. The MB populations were kept at 25°C under constant light in 2×250 ml bottles containing standard sugar/yeast/agar (SYA) medium [38]. Densities were approximately 300–350 flies per bottle. The size difference between North and South were still maintained under laboratory condition.

The MB populations used in the tiling array and subsequent real time PCR analysis were from the northern (NMB) and southern (SMB) ends of the cline. The *p{UAS}* transgenic lines were obtained from the Bloomington Stock Center (http://flystocks.bio.indiana.edu/) or the Exelixis Collection at Harvard (https://Drosophila.med.harvard.edu/). The *UAS-RNAi* strains were from the Vienna *Drosophila* RNAi Center (http://stockcenter.vdrc.at/). All fly stocks were reared at 25°C at 40%–50% humidity.

### Collection of larvae

Since we want to compare the gene expression profile of the different geographic populations, and gene expression changes very rapidly during larval development, larvae of different populations were reared under identical conditions and collected at the same age. Eggs and larvae for all populations within a replicate were collected within one hour. Throughout sample collection, paired sets of southern and northern samples were handled together, to minimise environmental variation.

Two hundred synchronized adult flies of each of the 20 MB populations were gathered to lay eggs on an agar base in a Petri dish with yeast. Larval samples were collected at 64 hr (second instar larvae) and 100 hr (third instar larvae) after egg-laying. At harvest, larvae were carefully removed from the medium with a spatula, snap-frozen in liquid nitrogen, and stored at −80°C until RNA extraction.

### RNA isolation and array hybridizations

Second and third instar larval samples of five SMB and five NMB populations (Table S2) representing 7 independent sampling points (4 South and 3 North) were chosen for microarray hybridizations (Affymetrix *Drosophila* tiling array 2.0R). We ensured that the chosen southern and northern samples were handled in parallel throughout sample preparations.

Preparation of RNA samples for the microarray analysis largely followed the Affymetrix manual. Briefly, samples were homogenized in 1 ml TRIzol reagent (Invitrogen, Carlsbad, CA, USA) in FastPrep tubes (Lysing Matrix D; Q-Biogene, Mrogan Irvine, CA, USA) using a bead mill (Hybaid RiboLyser; Hybaid, Teddington, UK). Total RNA was isolated using TRIzol reagent and the RNeasy (Qiagen, Hilden Germany) kit, following the manufacturers' instruction. The mRNA fraction from total RNA was first purified and enriched by rRNA reduction using the RiboMinusTM Transcriptome Isolation Kit (Invitrogen P/N K1550-02). Isolated mRNA was converted into double-stranded (ds) DNA using the GeneChip® WT Amplified Double-Stranded cDNA Synthesis Kit and amplified by in vitro transcription into cRNA using the same kit [39]. The fragmented cRNA samples were labeled, hybridized and scanned by the Affymetrix microarray service at the laboratory of Professor Julian Dow (University of Glasgow).

### Tiling array data analysis

The data set consisted of five biological replicates of four conditions (North 2^nd^ instar larvae, South 2^nd^ instar larvae, North 3^rd^ instar larvae, South 3^rd^ instar larvae) hybridized on Affymetrix *Drosophila* Genome tiling arrays 2.0. The probe sequences were aligned against the whole *D. melanogaster* genome (release 5.5, Feb 2008) [40], allowing for one alignment error on either strand. 2794677 probes were retained (out of 2877099 locations, 2877067 unique sequences), i.e. those probes mapping to a single location in the genome. A probe was labelled as intergenic if it matched a genomic region outside of all gene boundaries. Otherwise, it was labelled either exonic if it matched at least partially an exon described/predicted in *D. melanogaster* genome release 5.5, or intronic if not.

DNA sequence variation between North and South might affect transcription analysis. We examined density plot of raw hybridization intensity and found no evidence of systematic bias favouring either end of the cline. Before normalization, we performed one round of background subtraction on an experiment and chromosome basis. The background was defined by the following procedure: for each probe, we took the measured intensities for the probe itself, and for the 500 probes on each side of the probe being considered. We then used the bottom 5 percentile value from these 1001 intensities to define the local background value. Finally, we used local regression [41] to smooth local background values. After background subtraction, we performed variance stabilization normalization using VSN [42]. Data normalization across the experiments, while adjusting for effects that arise from variation in the microarray technology, will also adjust for systematic sequence bias (Figure S1).

Differential expression was assessed in two steps: first, we used the hierarchical Bayesian model implemented in Limma [18] on a probe by probe basis. Then we considered that a probe could be differentially expressed in three different scenarios: (1) short, when the probe's P value is below 0.00001, (2) medium, when the geometric average of the probe's P value and the two probes upstream and downstream of it is below 0.01, or (3) long, when the geometric average of P values for 21 probes (10 probes on each side) weighted by a Gaussian function of variance 5 is below 0.1.

### Catmap analysis

For our Catmap analysis, a ranked gene list based on the Bayesian *t* statistic from Limma was used as input. The Wilcoxon rank sum was used to generate a score based on the sum of the rankings of all genes with a particular functional annotation, and the significance of that score (the P value) was calculated based on a random gene-rank distribution [20]. Gene categories were considered significantly differentially regulated at FDR (false discovery rate) <0.1.

### Quantitative real-time PCR

Total RNA was extracted as above for all 20 cline end MB populations (Table S2 and S3). The mRNA was reverse transcribed using oligo-dT primer and the SuperScript® II system (Invitrogen). Quantitative PCR was performed using the PRISM 7000 Sequence-Detection System (Applied Biosystems), with SYBR Green (Molecular Probes) as the fluorescent dye, following the manufacturers' instructions. qRT-PCR primers were designed for the 30 candidate genes (Table S4). Primers were optimized (Advanced Biosystems procedures), and relative quantities of transcripts were determined (relative standard curve method) and normalized to the housekeeping gene *actin5C*.

### Candidate gene RNAi and over-expression analysis

We mis-expressed 11 candidate genes and the transcription factor *srp* using p{UAS} or UAS-RNAi lines in which the GAL4 enhancer was driven via the ubiquitously expressed *daughterless* promoter. Strains were compared on the *w^Dah^* (*Wolbachia -*) genetic background after backcrossing for eight generations. Adults were allowed to mate and lay eggs for 4 hours on a coloured agar medium to improved egg visibility. To control for larval density, 50 eggs were transferred into individual vials containing 7 mL of standard fly medium. Wing size (right side) was determined as previously described [4] after capturing the images under a compound microscope with a camera on at least 30 males and 30 females per cross. The coordinates of five landmarks around the external margins of each wing were recorded and measured using ImageJ (http://rsbweb.nih.gov/ij/). Wing sizes were recorded in pixels and converted to mm^2^.

### Clinal expression analysis

*Drosophila melanogaster* mass bred populations were established using flies collected along the east coast of Australia in March/April 2008 (Table S5). Adults were allowed to mate for 4 hours and 50 eggs were spotted onto each food vial per population. Third instar larvae were harvested 96 hrs post hatching and stored at -70°C until RNA isolation.

Total RNA from 10 larvae per population was isolated using TRIzol® Reagent (Invitrogen). Total RNA was dissolved in 100 µL water and contaminating genomic DNA was removed using RQ1 RNase-Free DNase (Promega). DNase treated total RNA was purified using RNeasy Mini Kit (Qiagen) and first strand cDNA was synthesized using SuperScript® III First-Strand Synthesis SuperMix (Invitrogen).

Real time PCR reactions were set up using the LightCycler® 480 High Resolution Melting Master (Roche). Real time results were normalized using a house keeping gene, *RpL11,* using the conventional ΔΔCt method to estimate relative expression. Cycling conditions were 95°C for 10 min followed by 50 cycles of 95°C for 10 seconds, 58°C for 15 seconds and 72°C for 15 seconds. Acquisition of data was carried out at each cycle immediately after the extension phase. The purity of the amplicons was verified by post-amplification T_m_ (melting temperature) analysis. Primers used for real time PCR are listed in Table S4.

### Sequence analysis of the srp gene

Isofemale lines of the 2008 clinal populations were used for a polymorphism survey of the *srp* gene. Populations were from Innisfail, Queensland (17°31′S, 146°01′E), Rockhampton, Queensland (23°08′S, 150°43′E), Maryborough, Queensland (25°32′S, 152°41′E), Ballina, New South Wales (28°45′S, 153°31′E), Port Macquarie, New South Wales (31.25°′S, 152°52′E), Crooked River Winery, New South Wales (34°44′S, 150°48′E), Melbourne, Victoria (37°43′S, 145°22′E) and Cygnet, Southern Tasmania (43°09′S, 147°07′E). Genomic DNA of 12 isofemale lines from each geographic locality was extracted using the Qiagen blood and tissue kits from 25–30 flies. PCR reactions were performed in a thermal cycler using Qiagen Taq polymerase following the manufacturer's protocol. The double-stranded PCR products were purified using exoSAP-IT (GE Healthcare). Purified PCR products were sequenced using an Applied Biosystems 3730XL 96-capillary automated DNA sequencer. Sequences were edited and assembled using Codon Code Aligner Software. ClustalW was used to align sequences for further analyses. Manual adjustments were made where necessary.

## Supporting Information

Figure S1 Comparison of density plot of normalized array hybridization intensity (log) at gene regions between northern and southern populations. (A) Whole genome level 2^nd^ instar larvae (B) Whole genome level 3^rd^ instar larvae stages (C) 3RP region 2^nd^ instar larvae (C) 3RP region 3^rd^ instar larvae.
(DOCX)

Table S1 Candidate genes significantly differentially expressed between North and South. Bold letters indicate genes that were subjected to mis-expression. ^$^ indicates p{UAS} line subsequently tested. ^£^ RNAi line tested. * fold changes estimated from real time PCR.^ &^ *D. melanogaster* genome release 5.5.
(DOCX)

Table S2 Populations used for tiling array and real time PCR.
(DOCX)

Table S3 Additional populations used for RT PCR.
(DOCX)

Table S4 Primers sequences of 29 candidate genes, *Nf1* and *srp.*
(DOCX)

Table S5 Latitudinal coordinates of sampled populations for clinal expression analysis.
(DOCX)
