# Supplementary material for: Genome-Wide Transcription Analysis of Clinal Genetic Variation in Drosophila
Source: PLoS One. 2012 Apr 13;7(4):e34620. doi: 10.1371/journal.pone.0034620 (PMC3326059; doi:10.1371/journal.pone.0034620)
Supplement: Figure S1 — Comparison of density plot of normalized array hybridization intensity (log) at gene regions between northern and southern populations. (A) Whole genome level 2nd instar larvae (B) Whole genome level 3rd instar larvae stages (C) 3RP region 2nd instar larvae (C) 3RP region 3rd instar larvae. (DOCX) [file pone.0034620.s001.docx]

Figure S1 Comparison of density plot of normalized array hybridization intensity (log) at gene regions between northern and southern populations. (A) Whole genome level 2^nd^ instar larvae (B) Whole genome level 3^rd^ instar larvae stages (C) 3RP region 2^nd^ instar larvae (C) 3RP region 3^rd^ instar larvae.
