# Supplementary material for: Genome-Wide Transcription Analysis of Clinal Genetic Variation in Drosophila
Source: PLoS One. 2012 Apr 13;7(4):e34620. doi: 10.1371/journal.pone.0034620 (PMC3326059; doi:10.1371/journal.pone.0034620)
Supplement: Table S1 — Candidate genes significantly differentially expressed between North and South. Bold letters indicate genes that were subjected to mis-expression. $ indicates p{UAS} line subsequently tested. £ RNAi line tested. * fold changes estimated from real time PCR. & D. melanogaster genome release 5.5. (DOCX) [file pone.0034620.s002.docx]

Table S1 Candidate genes significantly differentially expressed between North and South. Bold letters indicate genes that were subjected to mis-expression. ^$^ indicates p{UAS} line subsequently tested. ^£^ RNAi line tested. * fold changes estimated from real time PCR. ^&^ *D. melanogaster* genome release 5.5.

| Gene | Chromosomal location^&^ | Molecular function | Fold Change^*^ |
| --- | --- | --- | --- |
|  | | | |
| **Up-regulated in South, both larval stages** | | | |
| CG11034 | 2L 5805479..5808858 | Dipeptidyl-peptidase activity; serine-type peptidase activity. proteolysis | 2.10 |
| **Jon44E^£^** | 2R 4578955..4579856 | serine-type endopeptidase activity, proteolysis | 2.96 |
| **CG6776^$^** | 3L 8511988..8513410 | glutathione transferase activity; glutathione dehydrogenase (ascorbate) activity; transferase activity, transferring sulfur-containing groups; pyrimidodiazepine synthase activity. metabolic process | 3.77 |
| **CG32073^$^** | 3L 11100128..11100457 | Unknown | 3.21 |
| **CG3984^$^** | 3R 10962884..10964626 | Unknown | 1.70 |
|  | | | |
| **Up-regulated in North, both larval stages** | | | |
| Cyp4p1 | 2R 5127548..5129642 | Electron carrier activity; heme binding; iron ion binding; monooxygenase activity | 6.98 |
| **elp1^$^** | 2R 653323..6536144 | Proteasome activator | 3.36 |
| **CG13905^£^** | 3L 902898..903614 | Unknown | 3.40 |
| CG34035 | 3R 13238810..13240253 | Unknown | 2.23 |
|  | | | |
| **Up-regulated in South, 2^nd^ instar** | | | |
| CG6912 | 3R 10960860..10962702 | Unknown | 1.43 |
| CG17752 | 3R 15435947..15438159 | Secondary active organic cation transmembrane transporter activity, transport | 3.60 |
| Lsd1 | 3R 19589579..19592388 | Lipid storage droplet-1 sequestering of lipi | 1.48 |
| CG10182 | 3R 19412929..19415537 | Transferase activity, transferring groups other than amino-acyl groups | 1.43 |
|  | | | |
| **Up-regulated in North, 2^nd^ instar** | | | |
| Cyp28d2 | 2L 5207308..5209345 | Electron carrier activity; heme binding; iron ion binding; monooxygenase activity | 4.95 |
| Uro | 2L 7780085..7781434 | Urate oxidase activity, allantoin biosynthetic process; purine base metabolic process | 2.26 |
| CG9259 | 2L 21089565..21090999 | Unknown | 5.10 |
| GstE7 | 2R 14294440..14295193 | Glutathione S transferase E7 | 3.00 |
| CG32074 | 3L 11125088..11125420 | Unknown | 6.03 |
| GstD2 | 3R 8197720..8198367 | Glutathione transferase activity; glutathione peroxidase activity | 2.12 |
| **GstD6^£^** | 3R 8202894..8203541 | Same as above | 1.50 |
| **CG5999^£^** | 3R 8567750..8569344 | Glucuronosyltransferase activity | 1.28 |
| **geko^$^** | 3R 21126633..21128012 | Sensory Perception | 1.80 |
| **CG31436^$^** | 3R 21129899..21131374 | Unknown | 2.45 |
|  | | | |
| **Up-regulated in North, 3^rd^ instar** | | | |
| **Ugt36Bc^£^** | 2L 16799029..16801585 | Glucuronosyltransferase activity, metabolic process | 1.50 |
| CG12934 | 2R 6536417..6537250 | Unknown | 1.30 |
| Cyp6a23 | 2R 10763338..10765159 | Electron carrier activity; heme binding; iron ion binding; monooxygenase activity | 3.12 |
| Cyp6a14 | 2R 4451849..4453442 | Same as above | 3.28 |
| Cyp304a1 | 3R 8789519..8791652 | Same as above | 1.40 |
| CG7069 | 3R 18198557..18201301 | Pyruvate kinase activity; magnesium ion binding; potassium ion binding | 2.24 |
