# Supplementary material for: Genome-Wide Transcription Analysis of Clinal Genetic Variation in Drosophila
Source: PLoS One. 2012 Apr 13;7(4):e34620. doi: 10.1371/journal.pone.0034620 (PMC3326059; doi:10.1371/journal.pone.0034620)
Supplement: Table S2 — Populations used for tiling array and real time PCR. (DOCX) [file pone.0034620.s003.docx]

Table S2 Populations used for tiling array and real time PCR

|  | STRAINS | ORIGIN | Latitude |
| --- | --- | --- | --- |
| SOUTHERN MASS BREDS | SMB1 | Spreyton Ayer Orchard, Tasmania | S 41° 13'  E 146° 20' |
|  | SMB4 | Grove-Hansen’s Orchard, Tasmania | S 42° 58'  E 147° 7' |
|  | SMB5, SMB6 | Hounville- Frankcombe, Tasmania | S 43° 1'  E 147° 2' |
|  | SMB7 | Hillwood-Miller’s, Tasmania | S 41° 14'  E 146° 59' |
| NORTHERN MASS BRED | NMB3, NMB4 | Innisfail, Queensland | S 17° 31'  E 146° 1' |
|  | NMB5, NMB6 | Cardwell, Queensland | S 18° 15'  E 146° 1' |
|  | NMB10 | Mossman, Queensland | S 16° 27'  E 145° 22' |
