# Supplementary material for: Genome-Wide Transcription Analysis of Clinal Genetic Variation in Drosophila
Source: PLoS One. 2012 Apr 13;7(4):e34620. doi: 10.1371/journal.pone.0034620 (PMC3326059; doi:10.1371/journal.pone.0034620)
Supplement: Table S3 — Additional populations used for RT PCR. (DOCX) [file pone.0034620.s004.docx]

Table S3 Additional populations used for RT PCR

|  | STRAINS | ORIGIN | Latitde |
| --- | --- | --- | --- |
| SOUTHERN MASS BREDS | SMB2 | Spreyton Ayer Orchard, Tasmania | S 41° 13'  E 146° 20' |
|  | SMB3 | Grove-Hansen’s Orchard, Tasmania | S 42° 58'  E 147° 7' |
|  | SMB8 | Hillwood-Miller’s, Tasmania | S 41° 14'  E 146° 59' |
|  | SMB9, SMB10 | Renelagh-Lucaston, Tasmania | S 43° 0'  E 147° 2' |
| NORTHERN MASS BRED | NMB1, NMB2 | Innisfail, Queensland | S 17° 31'  E 146° 1' |
|  | NMB7, NMB8 | Cardwell, Queensland | S 18° 15'  E 146° 1' |
|  | NMB9 | Ingham, Queensland | S 17° 31'  E 146° 3' |
