# Supplementary material for: Genome-Wide Transcription Analysis of Clinal Genetic Variation in Drosophila
Source: PLoS One. 2012 Apr 13;7(4):e34620. doi: 10.1371/journal.pone.0034620 (PMC3326059; doi:10.1371/journal.pone.0034620)
Supplement: Table S4 — Primers sequences of 29 candidate genes, Nf1 and srp. (DOCX) [file pone.0034620.s005.docx]

Table S4 Primers sequences of 29 candidate genes, *Nf1* and *srp*

| Cyp6a23_F | AGGTCGTCATGGAAACCCTT |
| --- | --- |
| Cyp6a23_R | TGTCTGGGTCATTCGCGTTA |
| Cyp4p1_F | ATTGCCAAAAGGAGGGACTT |
| Cyp4p1_R | AGCGGTGCTTGAACTTTTGT |
| CG13905_F | AACTTTGCCTTGGAGCTGAA |
| CG13905_R | CAGCTTCTCGATGTTGTCCA |
| Ugt36Bc_F | GCAGAACTTTGCCAGCACTT |
| Ugt36Bc_R | TGCTCCAAAGACGGTCATAG |
| CG34035-F | aagacgtgcctccaagctaa |
| CG34035-R | cgtttgccacctccacttat |
| StIP-F | ACGCAAACACAAGCTCACTG |
| StIP-R | CGACAACGAAATCCAGACCT |
| CG32074-F | TCGCAGCCAACTATCTTCCT |
| CG32074-R | CAGGCAGTCGAAACGAAAG |
| GstD2_F | GGATCGGATGAGGACTTGAA |
| GstD2_R | GGAAGGTGTCGAGAAATCCA |
| GstD6_F | AAACCCTCAGCACACGATTC |
| GstD6_R | CCGAGTTTCCCAAATAACGA |
| CG5999_F | GGTACAAGGAAGTTTATGGCAA |
| CG5999_R | CAACGATGTGTTCTTGAGCAT |
| CG13659_F | TCACGAGCAACCTGAATATCT |
| CG13659_R | CCCGGGCATTTCACACAAT |
| Cyp28d2_F | TCACTGTCCAGGAATGCGAT |
| Cyp28d2_R | CTCAAGATTTCCACCAGAGCT |
| CG9259_F | GGAACAGAGAACGGGACAAA |
| CG9259_R | GGCATTCTCCACCACTGATT |
| GstE7_F | TGATTCCCAAGGAGCGTTAC |
| GstE7_R | CCAGCAAGGAATTTCTCCAG |
| Uro-F | GGTTATAAGGAGAACAGATCCCA |
| Uro-R | CGAGGATTGGGTCGTCTTCA |
| CG31436-F | CAACACAAGTACTACGAGTTCT |
| CG31436-R | GTCAACGGACTTATCCCTCAAT |
| Cyp6a14_F | TGGAGTGCAACAGTCTCCAG |
| Cyp6a14_R | TGAATATTTCGCGACCCTTC |
| CG12934_F | GAAGTCCAACAGCAGCATCA |
| CG12934_R | TCCACTGCTCCCAAAGAAAC |
| Cyp304a1_F | GGATACCCAAGGACACCATT |
| Cyp304a1_R | CTCCGGATCCGACCAAATA |
| CG7069_F | GCTGAAAAGGAGGAATGCAG |
| CG7069_R | GGCCAGTTTTTGCTTCTGAC |
| CG11034_F | GGCTGTACTATGATACGA |
| CG11034_R | AACACGCTGCTCTCGTTGTA |
| Jon44E_F | CAGCGACATGATCCAACATC |
| Jon44E_R | TCAGAGCGATGTCGTTGTTC |
| CG6776_F | GTCGCTGATTATTGCCGAGT |
| CG6776_R | AACGGATTCTCCGGGTACTT |
| CG32073-F | ACTGGCACCTCGACTACGTT |
| CG32073-R | TGGTGGTTGTGGTAGTGGTG |
| CG3984_F | AGCAGCCCCAGAACTTCTA |
| CG3984_R | AGTACGAGGAGCAGGTCGA |
| CG6912_F | AGTGGTCATGCCGCCAAAT |
| CG6912_R | GAGCAATCCAAAATGTCGCTCT |
| Lsd-1-F | GTGCAGATGAACCCGAAAAT |
| Lsd-1-R | GCACAATCAACTGCTCCAGA |
| CG17752_F | TGCACAAGCAACTACCACAA |
| CG17752_R | TGCGTGAAAAGCAAGGGAA |
| CG10182_F | CGAACTACACCATTATGCTCA |
| CG10182_R | GCCTCAATTACCAAGTGCAT |
| CG9466_F | TTGTGCAGACATCGCTGATCA |
| CG9466_R | TCCGTTCATTTCAACCCTCTT |
| Syt-F | GAAGGGTGTCGACATGAAGT |
| Syt_R | ATCAGGCTGAACTTTCTCCTT |
| Dmds_srp_rt01 | AGCAGCCAGATGGATGTCAATG |
| Dmds_srp_rt02 | TGCTGGTACTGTGGAAGCTGTTG |
| RpL11_f | CGATCTGGGCATCAAGTACGAT |
| RpL11_r | TTGCGCTTCCTGTGGTTCAC |
