# Supplementary material for: Genome-Wide Transcription Analysis of Clinal Genetic Variation in Drosophila
Source: PLoS One. 2012 Apr 13;7(4):e34620. doi: 10.1371/journal.pone.0034620 (PMC3326059; doi:10.1371/journal.pone.0034620)
Supplement: Table S5 — Latitudinal coordinates of sampled populations for clinal expression analysis. (DOCX) [file pone.0034620.s006.docx]

Table S5 Latitudinal coordinates of sampled populations for clinal expression analysis

| Population | Latitude |
| --- | --- |
| Pop's Place | 43° 09’ |
| Miller's orchard | 41° 14’ |
| Melbourne | 37° 43’ |
| Metung | 37° 53’ |
| Tilba Winery | 36° 17’ |
| Crooked River Winery | 34° 44’ |
| Port Macquarie | 31° 25’ |
| Coffs Harbour | 30° 22’ |
| Maryborough | 25° 32’ |
| Finch Hatton/Eungella | 21° 08’ |
| Bowen | 19° 58’ |
| Cardwell | 18° 12’ |
| Innisfail | 17° 31’ |
| Atherton/Cairns | 17° 12’ |
| Cape Tribulation | 16° 15’ |
